# The Effect on Mortality of Bacterial Co-Infections on Critically Ill Patients with Community-Acquired COVID-19 and Influenza Pneumonia: A Systematic Review

**DOI:** 10.3390/v17060851

**Published:** 2025-06-16

**Authors:** Apostolos A. Menis, Efrosyni Gerovasileiou, Konstantinos Mantzarlis, Efstratios Manoulakas, Konstantina Deskata, Vasileios Vazgiourakis, Demosthenes Makris, George Dimopoulos

**Affiliations:** 1 Intensive Care Unit, University Hospital of Larisa, 41334 Larisa, Greece; gerovasileiou@yahoo.com (E.G.); mantzk@outlook.com (K.M.); stratosfox@hotmail.com (E.M.); konstadv@gmail.com (K.D.); vasvazg@yahoo.com (V.V.); appollon7@hotmail.com (D.M.); 2 Intensive Care Unit, Evgenidio Hospital, National and Kapodistrian University of Athens, 11528 Athens, Greece; gdimop@med.uoa.gr

**Keywords:** mortality, critical care, virus, pneumonia, co-infection

## Abstract

**Background:** Bacterial co-infections in patients with viral pneumonia might increase mortality. In this study we aimed to evaluate their effect on the mortality of critically ill patients with viral pneumonia. **Methods:** A systematic search was conducted in PubMed, Web of Science, Scopus and Cochrane from inception until 30 March 2025. We included studies comparing the effect on mortality of bacterial co-infections in critically ill patients with viral pneumonia. The risk of bias was assessed by the Newcastle–Ottawa Scale. **Results:** From 3643 studies, 10 were included in our study with a total of 2862 COVID-19 patients and 4573 influenza patients. Seven studies were retrospective and three prospective. In total, 359/2862 of the COVID-19 and 904/4573 of the influenza patients were co-infected. Co-infections increased mortality in five out of the six studies evaluating COVID-19 patients and in two out of the eight studies evaluating influenza patients. **Conclusions:** The majority of the included studies were retrospective, which may limit the accuracy of these results. The exclusion of non-English literature may have led to the omission of relevant data. Based on our results, the impact of bacterial co-infection may be more pronounced in patients with COVID-19 pneumonia admitted to the ICU than in patients with influenza pneumonia.

## 1. Introduction

Severe pneumonia is associated with a mortality rate of up to 38% [1], making it a leading cause of death among patients admitted to the intensive care unit (ICU). Viruses such as SARS-CoV-2 and H1N1 are common causative factors of severe pneumonia, accounting for a significant proportion of ICU admissions [2]. In these patients, bacterial co-infections are frequently reported and are thought to contribute to worse clinical outcomes [3,4,5]. Previous studies assessing bacterial co-infection in viral pneumonia have reported conflicting results regarding its impact on mortality in the general patient population [5,6,7,8,9,10,11]. Furthermore, most available studies have focused on hospitalized patients broadly, with few specifically investigating outcomes in critically ill patients admitted to the ICU. Critically ill patients represent a distinct population, characterized by higher severity of illness, greater need for invasive mechanical ventilation and worse outcomes. These factors may alter the clinical impact of bacterial co-infections. As such, the lack of systematic reviews focused on ICU patients is detrimental.

Given the limited and ambiguous evidence, particularly in critically ill adults, there is a need for a systematic synthesis of the available data to better understand the role of bacterial co-infection in this high-risk population. Therefore, the aim of this systematic review was to evaluate the impact of bacterial co-infection on mortality in critically ill patients with viral pneumonia.

## 2. Materials and Methods

This systematic review was conducted according to the criteria established by the PRISMA guidelines [12] and was based on a predefined protocol agreed upon by all authors. Although it was not prospectively registered in PROSPERO, all methodological steps were transparently reported.

The studies that were included in this systematic review were about the effect on mortality of bacterial co-infections in critical care patients with viral severe pneumonia.

Studies were excluded if they were classified as: case reports, case series, narrative reviews, letters to the editor, correspondences, literature reviews, scoping reviews, systematic reviews, meta-analyses, books, abstracts or unpublished manuscripts; or if they included patients of pediatric ICUs. Bacterial co-infection was defined as the presence of a pathogenic isolate from sterile site cultures taken within 48-h of admission (ICU or hospital).

A systematic search was conducted on PubMed, Scopus, Web of Science and Cochrane from inception to 30 March 2025. The search was conducted without any additional filters. The search strategy followed in each database are the following:

- PubMed: (ARDS OR “Acute Respiratory Distress Syndrome” OR “Respiratory failure” OR “Severe Pneumonia” OR SARS OR “Pneumonia” [MESH] “Severe acute respiratory syndrome” OR “Respiratory Distress Syndrome” [Mesh] OR “adult hyaline membrane disease” OR “postperfusion lung” OR “pump lung” OR “shock lung” OR “adult respiratory insufficiency syndrome” OR “SARS” OR MERS OR “middle east respiratory syndrome” OR Virus OR Viral OR “Virus Diseases” [MeSH]) AND (“Intensive Care Unit” OR ICU OR “Critical Care” OR “Intensive Care Units” [MeSH]) AND (Co-infection OR “Bacterial Co-Infection” OR “Co infection” OR “coinfection” OR superinfection OR “Coinfection” [MeSH]) AND (Mortality OR Death OR “Mortality” [MeSH])
- Scopus: (TITLE-ABS-KEY (ards OR “acute respiratory distress syndrome” OR “respiratory failure” OR “severe pneumonia” OR “severe acute respiratory syndrome” OR sars OR “respiratory distress syndrome” OR “pneumonia” OR “adult hyaline membrane disease” OR “postperfusion lung” OR “pump lung” OR “shock lung” OR “adult respiratory insufficiency syndrome” OR mers OR “middle east respiratory syndrome” OR virus OR viral OR “virus diseases”) OR AUTHKEY (ards OR “acute respiratory distress syndrome” OR “respiratory failure” OR “severe pneumonia” OR “severe acute respiratory syndrome” OR sars OR “respiratory distress syndrome” OR “pneumonia” OR “adult hyaline membrane disease” OR “postperfusion lung” OR “pump lung” OR “shock lung” OR “adult respiratory insufficiency syndrome” OR mers OR “middle east respiratory syndrome” OR virus OR viral OR “virus diseases”)) AND (TITLE-ABS-KEY (“intensive care unit” OR icu OR “critical care” OR “intensive care units”) OR AUTHKEY (“intensive care unit” OR icu OR “critical care” OR “intensive care units”)) AND (TITLE-ABS-KEY (“co-infection” OR “bacterial co-infection” OR “co infection” OR coinfection OR superinfection) OR AUTHKEY (“co-infection” OR “bacterial co-infection” OR “co infection” OR coinfection OR superinfection)) AND (TITLE-ABS-KEY (mortality OR death OR fatality) OR AUTHKEY (mortality OR death OR fatality))
- Web of Science: TS = (ARDS OR “acute respiratory distress syndrome” OR “respiratory failure” OR “severe pneumonia” OR “severe acute respiratory syndrome” OR SARS OR “respiratory distress syndrome” OR “pneumonia” OR “adult hyaline membrane disease” OR “postperfusion lung” OR “pump lung” OR “shock lung” OR “adult respiratory insufficiency syndrome” OR MERS OR “middle east respiratory syndrome” OR virus OR viral OR “virus diseases”) AND TS = (“intensive care unit” OR ICU OR “critical care” OR “intensive care units”) AND TS = (“co-infection” OR “bacterial co-infection” OR “co infection” OR coinfection OR superinfection) AND TS = (mortality OR death OR fatality)
- Cochrane: #1 ARDS: ti,ab,kw, #2 “acute respiratory distress syndrome”: ti,ab,kw, #3 “respiratory failure”: ti,ab,kw, #4 “severe pneumonia”: ti,ab,kw, #5 SARS: ti,ab,kw, #6 “severe acute respiratory syndrome”: ti,ab,kw, #7 “respiratory distress syndrome”: ti,ab,kw, #8 pneumonia: ti,ab,kw, #9 “adult hyaline membrane disease”: ti,ab,kw, #10 “postperfusion lung”: ti,ab,kw, #11 “pump lung”: ti,ab,kw, #12 “shock lung”: ti,ab,kw, #13 “adult respiratory insufficiency syndrome”: ti,ab,kw, #14 MERS: ti,ab,kw, #15 “middle east respiratory syndrome”: ti,ab,kw, #16 virus: ti,ab,kw, #17 viral: ti,ab,kw, #18 “virus diseases”: ti,ab,kw, #19 (#1 OR #2 OR #3 OR #4 OR #5 OR #6 OR #7 OR #8 OR #9 OR #10 OR #11 OR #12 OR #13 OR #14 OR #15 OR #16 OR #17 OR #18), #20 “intensive care unit”: ti,ab,kw, #21 ICU: ti,ab,kw, #22 “critical care”: ti,ab,kw, #23 “intensive care units”: ti,ab,kw, #24 (#20 OR #21 OR #22 OR #23), #25 “co-infection”: ti,ab,kw, #26 “bacterial co-infection”: ti,ab,kw, #27 “co infection”: ti,ab,kw, #28 coinfection:ti,ab,kw, #29 superinfection: ti,ab,kw, #30 (#25 OR #26 OR #27 OR #28 OR #29), #31 mortality: ti,ab,kw, #32 death:ti,ab,kw, #33 fatality: ti,ab,kw, #34 (#31 OR #32 OR #33), #35 (#19 AND #24 AND #30 AND #34).

Each record was screened independently by two reviewers, and disagreements were solved by a third member. We limited our review to only English language articles due to technical restrictions. No automation tools, machine learning classifiers/algorithms, crowdsourcing or datasets of already-screened records were used within the overall selection process. Data from each report were collected by two independent reviewers and any disagreement between the two data collectors was solved by a third member.

The primary outcome of this systematic review was the effect on mortality of bacterial co-infections on critically ill patients with community-acquired viral pneumonia. The secondary outcome was the microbiology and prevalence of the bacterial co-infections in this population.

Risk of bias assessment was conducted using the Newcastle–Ottawa Scale (NOS) assessment for observational studies. NOS studies and evaluates the risk of bias across three domains: patient selection, comparability and outcome. Two reviewers worked independently to assess the risk of bias and any disagreement between the two reviewers was solved by a third member. Extracted data included the first author, year of publication, study design, co-infection definition, population size, study setting, sample size, rate of bacterial co-infection, mortality outcomes, and effect estimates (odds ratios with corresponding 95% confidence intervals, when reported). For each included study, we also collected information on the bacterial pathogens isolated among co-infected patients, along with details on the types of samples collected and the diagnostic methods used for detecting COVID-19, influenza, and bacterial co-infections. When pathogen counts were reported at the species level, these were aggregated to the genus level to allow consistent pooling across studies. Studies that reported only the most frequent pathogen without providing full organism counts were excluded from the microbiological analysis. We calculated the total number and percentage of each bacterial genus separately for COVID-19 and influenza patients. Additionally, we extracted data from each study on the type of ventilatory support provided to patients during their ICU stay, when reported. Subgroup analyses were conducted for microbiological data, stratifying bacterial pathogens by viral etiology (COVID-19 vs. Influenza) and by timing of co-infection (<48 h from ICU or hospital admission).

When odds ratios were not directly reported, we calculated them from raw outcome data using standard formulas for 2 × 2 contingency tables. We performed a narrative synthesis of the findings, summarizing the odds ratios for mortality across studies without statistical pooling or meta-analysis. The principal summary measure was the odds ratio (OR) with 95% confidence intervals. We did not assess reporting bias (e.g., publication bias), as no meta-analysis was conducted. Similarly, we did not perform sensitivity analyses or heterogeneity testing, nor did we formally assess the certainty of the evidence using GRADE or comparable methods. To visually summarize and compare effect estimates, we produced descriptive forest plots displaying the individual odds ratios and corresponding 95% confidence intervals for each study. No pooled effect estimate was calculated or displayed. Forest plots were generated using R version 4.3.2 (Rstudio 2024.04.2 Build 764) and the meta package for R [13], with odds ratios presented on a logarithmic scale to maintain symmetry around the null value (OR = 1). Separate plots were created for COVID-19 and influenza studies to facilitate comparison.

## 3. Results

### 3.1. Study Selection

**Figure 1:**
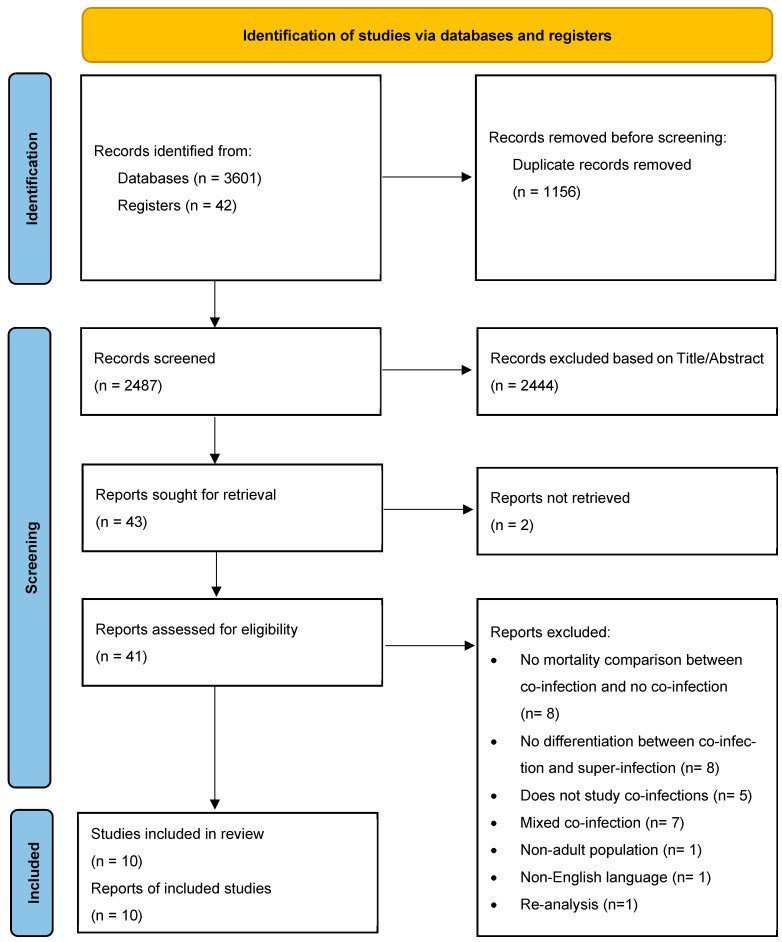
Flow chart of the study selection.

Based on the search strategy used 3601 articles from the databases and 42 from the Cochrane Clinical trials registry were found, and from these 1156 duplicate articles were removed. From the remaining 2487 articles, 2444 were excluded, based on the title and abstract, and the remaining 43 reports were sought for retrieval. The flow chart of the study selection is depicted in Figure 1.

From the 43 reports initially sought, 41 were retrievable. From these 41 studies, 31 were excluded. Eight studies were excluded as they did not compare the outcome of mortality between co-infected and not co-infected patients [14,15,16,17,18,19,20,21]; eight studies were excluded as they did not differentiate between co-infections and super-infections [22,23,24,25,26,27,28,29]; five studies were excluded as they did not study co-infections [30,31,32,33,34]; seven studies were excluded as the co-infection was of mixed causes (viral, bacterial, fungal) [35,36,37,38,39,40,41]; one study was excluded as it also included a pediatric population [4]; one study was excluded as it was written in a non-English language [42]; and one was excluded as it was a re-analysis of an included study [3].

### 3.2. Study Characteristics

Ten studies were included in this systematic review [43,44,45,46,47,48,49,50,51,52]. Seven were retrospective observational studies [43,44,45,46,47,48,49] while three where prospective observational studies [50,51,52]. Four studies included only patients with severe pneumonia due to influenza [45,46,51,52], two studies included only patients with severe pneumonia due to COVID-19 [44,50] and four studies included both cohorts [43,47,48,49]. Their key characteristics are displayed in Table 1. In total, the prevalence of bacterial co-infections in COVID-19 patients was 12.51% (359/2862 patients) while in influenza patients it was 19.8% (904/4573 patients). Moreover, five studies were multicentered [46,47,49,51,52]. Moreover, bacterial co-infection was defined as bacterial infection diagnosed <48 h from hospital admission in five studies [43,44,46,50,51], as bacterial infection diagnosed <48 h from ICU admission in four studies [45,48,49,52] and as bacterial infection diagnosed <48 h from intubation in one study [47].

### 3.3. Risk of Bias

The risk of bias assessment using the Newcastle–Ottawa Scale indicated that nine studies [43,44,45,46,47,48,49,51,52] achieved the maximum score of 9/9, reflecting low risk of bias across all assessed domains. One study [50] scored 7/9, as it did not include any adjustment for confounding factors, indicating moderate risk of bias. The risk of bias assessment of the included studies is depicted in Table 2.

### 3.4. Results of Individual Studies

Co-infections increased the mortality in five [44,47,48,49,50] out of the six [43,44,47,48,49,50] studies evaluating COVID-19 patients, and in two [49,51] out of the seven [45,46,47,48,49,51,52] studies evaluating influenza patients. In Martin-Loeches et al. [51], this association was statistically significant, while Chu et al. [49] observed a similar trend that did not reach statistical significance. In one study, there were no data regarding the number of deaths from bacterial co-infections in patients with influenza [48].

In those studies that defined co-infection as <48 h from hospital admission, an increase in mortality related to co-infections was shown in two [44,50] out of three studies evaluating COVID-19 patients, and in one [51] out of three evaluating influenza patients. Meanwhile, in those that defined co-infection as <48 h from ICU admission, an increase in mortality related to co-infections was shown in three out of the three [47,48,49] studies evaluating COVID-19 patients and in one [49] of the four studies [45,47,49,52] evaluating influenza patients, without, however, reaching statistical significance. The results are depicted in Table 3.

**Figure 2:**
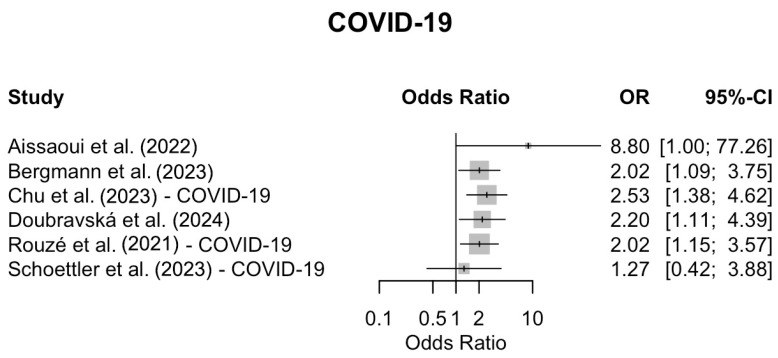
Forest plot displaying the odds ratios (ORs) and 95% confidence intervals (CIs) for mortality associated with bacterial co-infection in individual studies of critically ill patients with COVID-19. No pooled effect estimate was calculated; the plot is presented for descriptive purposes only. Odds ratios are shown on a logarithmic scale [43,44,47,48,49,50].

**Figure 3:**
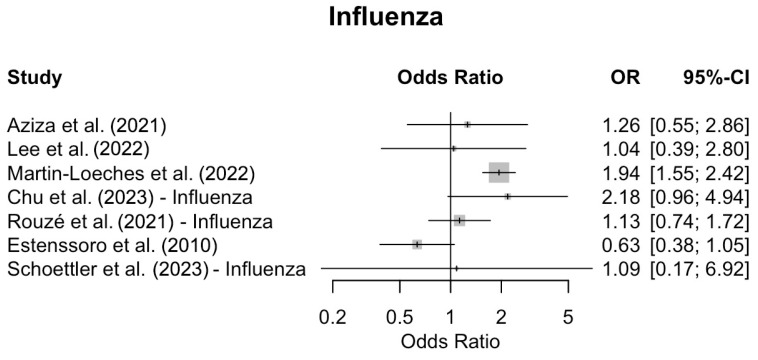
Forest plot displaying the odds ratios (ORs) and 95% confidence intervals (CIs) for mortality associated with bacterial co-infection in individual studies of critically ill patients with influenza. No pooled effect estimate was calculated; the plot is presented for descriptive purposes only. Odds ratios are shown on a logarithmic scale [43,45,46,47,49,51,52].

Forest plots were generated to visually summarize the individual study findings. Figure 2 shows the odds ratios and 95% CIs for COVID-19 studies, while Figure 3 presents the corresponding data for influenza studies. No meta-analysis was performed; the plots are presented for descriptive purposes only, with odds ratios displayed on a logarithmic scale to facilitate comparison.2

Moreover, from the studies that defined bacterial co-infection as those diagnosed within 48 h from hospital admission [43,44,46,50,51], data for time from diagnosis to ICU admission were available only in two [44,50]. In the study by Doubravská et al., the timeframe was <48 h while in the study by Aissaoui et al. the timeframe was 4 (2–6) days.

The pooled analysis of bacterial pathogens identified in co-infected patients showed that the most frequently isolated genera among all COVID-19 patients were *Staphylococcus aureus* (n = 66, 10.8%), *Streptococcus* spp. (n = 33, 5.4%), and *Klebsiella* spp. (n = 25, 4.1%). In influenza patients, the most common pathogens were *Streptococcus* spp. (n = 313, 40.1%), *Staphylococcus aureus* (n = 120, 15.4%), and *Pseudomonas aeruginosa* (n = 59, 7.6%). In COVID-19 patients with infections identified within 48 h of ICU admission, the most common pathogens were *S. aureus* (n = 50, 10.0%), *Streptococcus* spp. (n = 32, 6.4%), and *Klebsiella* spp. (n = 19, 3.8%). In contrast, among those identified within 48 h of hospital admission, *S. aureus* remained dominant (n = 16, 14.0%), followed by *Klebsiella* spp. (n = 6, 5.3%) and *Proteus* spp. (n = 4, 3.5%). For influenza patients, the most frequently isolated genera within 48 h of ICU admission were *Streptococcus* spp. (n = 63, 32.6%), *S. aureus* (n = 53, 27.5%), and *Haemophilus influenzae* (n = 19, 9.8%). Among those with infections diagnosed within 48 h of hospital admission, *Streptococcus* spp. remained the most prevalent (n = 250, 41.9%), followed by *S. aureus* (n = 67, 11.2%) and *Pseudomonas aeruginosa* (n = 59, 9.9%). From the 10 included studies, only six reported complete microorganism counts [43,44,46,47,48,51]. A complete summary of bacterial co-infections isolated by subgroup is presented in Table 4.

Nasopharyngeal swabs were used for viral detection in six studies [43,44,46,47,50,51], with PCR or RT-PCR performed in all studies. Respiratory cultures were conducted in eight studies [44,45,46,47,48,49,50,51], blood cultures in eight studies [44,45,46,47,48,49,50,51], and pleural fluid cultures in two studies [49,51]. Antigen testing for Streptococcus pneumoniae and Legionella pneumophila was performed in three studies [47,48,50]. One study reported PCR for bacterial nucleic acid and serological testing for Mycoplasma and Chlamydophila [50]. Two studies did not report bacterial testing methods [43,52] (Table 5).

## 4. Discussion

In this systematic review, 10 studies evaluating the effect of bacterial co-infection on the mortality of critically ill patients with viral pneumonia were included. Bacterial co-infections were a cause of increased mortality in five [44,47,48,49,50] out of the six [43,44,47,48,49,50] studies evaluating COVID-19 patients and in two [49,51] out of the seven [45,46,47,48,49,51,52] studies with influenza patients. To our knowledge, this is the first systematic review that assesses the impact of bacterial co-infection on the mortality of critically ill patients with viral pneumonia.

Based on our findings, bacterial co-infection was associated with increased mortality in COVID-19 patients in the majority of included studies, with all [44,47,48,49,50] except one [43] having a positive correlation between bacterial co-infection and mortality, whereas in influenza patients, mortality was largely unaffected, with two [49,51] out of the seven [45,46,47,48,49,51,52] studies reporting an increase in mortality. Notably, the proportion of patients requiring invasive mechanical ventilation varied substantially across studies, ranging from 42% to 100%. This variability reflects differing degrees of illness severity among study populations, complicating the safe generalization of results. Interestingly, in the COVID-19 study [43] where mortality was not associated with co-infections, patients had higher SOFA scores and a greater need for mechanical ventilation compared to the other studies [44,47,48,49,50], highlighting, perhaps, the fact that in patients with increased initial disease severity, co-infections do not alter the outcome. Previous studies have evaluated the impact of bacterial co-infection on mortality in COVID-19 [7,53,54,55,56,57,58] and influenza patients [6,11,56,58,59,60]. Among seven studies assessing COVID-19 patients [7,53,54,55,56,57,58], six identified bacterial co-infection as an independent predictor of mortality, while one out of the four studies on influenza patients reported a similar association [60]. Furthermore, two meta-analyses examining bacterial co-infection in influenza patients found an increased mortality risk in co-infected individuals [6,11]. However, none of these prior studies focused exclusively on critically ill adults, and most included pediatric populations, limiting direct comparisons to our review. Nevertheless, drawing firm conclusions remains difficult, as only three of the included studies enrolled a substantial number of critically ill patients. The small sample sizes and heterogeneity (e.g., type of ventilation, co-infection definition) across the remaining studies limit the strength of the overall evidence and highlight the need for larger, high-quality investigations focused specifically on ICU populations.

Furthermore, the prevalence of bacterial co-infection was higher in influenza patients than in COVID-19 patients. Several studies in the literature have investigated the prevalence of bacterial co-infection in COVID-19 [7,8,9,25,53,54,55,56,61,62] and influenza patients [6,10,25,56,59,60,61], reporting wide variability in their findings. In COVID-19 patients, reported prevalence ranged from 2.5% to 33.3%, while two prior meta-analyses estimated rates of 3.5% [9] and 7% [7], respectively. Notably, the meta-analysis by Lansbury et al. [7] reported a prevalence of 14% among ICU patients, which closely aligns with our findings. In influenza patients, reported prevalence ranged from 10.3% to 35.2%, with one study reporting rates as high as 55.6% among critically ill patients [59]. Three prior meta-analyses [6,10,11] reported prevalence estimates between 11% and 35% findings consistent with our results. One study [58] reported a higher rate of co-infections in COVID-19 patients compared to influenza, and a longer time from admission to bacterial growth in COVID-19 (4 [1,2,3,4,5,6,7,8] vs. 1 [1,2,3] days). However, this study included both early (<48 h) and late (>48 h) infections and was not limited to critically ill patients, which may explain the higher co-infection rate.

Finally, in the influenza group the most common pathogens in critical care patients with bacterial co-infection were *S. pneumoniae*, *S. aureus* and *P. aeruginosa.* These results are in accordance with previous meta-analyses evaluating bacterial co-infection in influenza patients in the general population [10,11]. However, in a previous study [46], an unusually high proportion of Gram-negative pathogens (65.7%) was reported along with a lower rate of streptococcal pneumonia (11.4%). The authors attributed this to a high burden of comorbidities such as diabetes, COPD, and liver cirrhosis in their ICU cohort, which may alter the expected pathogen distribution. Moreover, in the critically ill COVID-19 patients with bacterial co-infections, the most common pathogen was *S. aureus*. Similar results have been reported by previous studies [8,25,54,62]. Notably, the sample location and bacterial isolation methods varied across studies, which may partly explain the heterogeneity in prevalence rates and bacterial isolates. In addition, differences in diagnostic approaches further contribute to discrepancies between studies and highlight the need for more standardized diagnostic protocols in co-infection research. Interestingly, even though there was an increased prevalence of bacterial co-infections in patients with influenza, there was not a trend for increased mortality compared to COVID-19 patients with co-infections. Our hypothesis is that this could be explained by the fact that the major cause of co-infections in the influenza population was *S. pneumoniae*, which is associated with lower mortality rates compared to *S. aureus* [63], possibly due to the existence of an antipneumonococcal vaccine [64]. It should be noted though, that as no meta-analysis was conducted, no pooled estimate of mortality can be given.

### 4.1. Limitations

Most of the included studies were retrospective cohort designs, increasing the risk of bias due to potential misclassification, missing data, and residual confounding inherent in the use of pre-existing clinical records. Although several studies used multivariable adjustment, the possibility of unmeasured confounding remains. Additionally, while all studies focused on critically ill patients, not all participants were intubated, leading to variability in disease severity across studies. This heterogeneity limits the comparability of outcomes and may have introduced clinical variability. We also excluded studies published in languages other than English, which may have introduced language bias and led to the omission of relevant data from non-English publications. Publication bias and small-study effects could not be formally assessed because no meta-analysis was performed. Although we conducted a comprehensive search across multiple databases without date restrictions, and followed PRISMA guidelines [12] for study selection, we cannot completely exclude the possibility that relevant studies, particularly older studies or those with inconsistent terminology, may have been missed. Lastly, differences in microbiological diagnostic methods across studies, including the use of different sample types and laboratory techniques, may have contributed to variability in the reported rates of bacterial co-infection. These factors collectively limit the generalizability and comparability of the findings.

### 4.2. Implications

This systematic review examined the impact of early bacterial co-infection on mortality in critically ill patients with community-acquired viral pneumonia due to either COVID-19 or influenza. While several studies reported an association between bacterial co-infection and increased mortality in COVID-19, this effect was less consistent in influenza cohorts. However, interpretation is limited by key constraints: only a few studies included a substantial number of ICU patients, and methodological heterogeneity—particularly in the definition of co-infection timing—complicates comparability. As a result, this review does not demonstrate a clear or consistent difference in the impact of bacterial co-infection between COVID-19 and influenza. The current evidence base remains too limited to support firm conclusions. Future research should prioritize prospective, multicenter studies using standardized definitions, and consistent diagnostic approaches. Studies stratifying outcomes by illness severity, including the need for invasive mechanical ventilation, are also needed to better understand the clinical impact of bacterial co-infection in severe viral pneumonia.

## Figures and Tables

**Table 1:** Key characteristics of the included studies.

Study	Country	Setting	Design	Study Period	Sample Size	Invasive Ventilation	Population
**<48 h from Hospital Admission**							
Aissaoui et al. (2022) [44]	Morocco	ICU (single center)	Retrospective cohort	2020–2021	155	42%	COVID-19
Doubravská et al. (2024) [50]	Czech Republic	ICU (single center)	Prospective cohort	2020–2022	171	69%	COVID-19
Lee et al. (2022) [46]	Taiwan	ICU (multicenter)	Retrospective cohort	2014–2018	117	63.3%	Influenza
Martin-Loeches et al. (2017) [51]	Spain	ICU (multicenter)	Prospective cohort	2009–2015	2901	53%	Influenza
Schoettler et al. (2023) [43]	Germany	ICU (single-center)	Retrospective cohort	2009–2022	190	78.1%	COVID-19, Influenza
**<48 h from ICU Admission**							
Chu et al. (2023) [49]	Hong Kong	ICU (multicenter)	Retrospective cohort	2015–2021	746	COVID-19: 43.4% Influenza: 53.1%	COVID-19, Influenza
Aziza et al. (2021) [45]	Canada	ICU (single center)	Retrospective cohort	2014–2019	130	83%	Influenza
Rouzé et al. * (2021) [47]	Europe	ICU (multicenter)	Retrospective cohort	2016–2020	1050	100%	COVID-19, Influenza
Bergmann et al. (2023) [48]	Germany	ICU (single center)	Retrospective cohort	2015–2022	328	No data	COVID-19, Influenza
Estenssoro et al. (2010) [52]	Argentina	ICU (multicenter)	Prospective cohort	2009	337	81%	Influenza

* Timing of co-infection defined as <48 h from endotracheal intubation.

**Table 2:** The risk of bias assessment of the included studies according to the Newcastle–Ottawa Scale assessment.

Study	S1:	S2:	S3:	S4:	C1:	O1:	O2:	O3:	Total
Aissaoui et al. [44]	1	1	1	1	2	1	1	1	9/9
Aziza et al. [45]	1	0	1	1	2	1	1	1	9/9
Lee et al. [46]	1	1	1	1	2	1	1	1	9/9
Martin-Loeches et al. [51]	1	1	1	1	2	1	1	1	9/9
Bergmann et al. [48]	1	1	1	1	2	1	1	1	9/9
Chu et al. [49]	1	1	1	1	2	1	1	1	9/9
Doubravská et al. [50]	1	1	1	1	0	1	1	1	7/9
Rouzé et al. [47]	1	1	1	1	2	1	1	1	9/9
Estenssoro et al. [52]	1	1	1	1	2	1	1	1	9/9
Schoettler et al. [43]	1	1	1	1	2	1	1	1	9/9

Abbreviations: S1: Representativeness; S2: Selection of non-exposed; S3: Ascertainment of exposure; S4: Outcome not present at start; C1: Comparability; O1: Assessment of outcome; O2: Adequate follow-up duration; O3: Adequacy of follow-up.

**Table 3:** Characteristics of the individual studies.

Study	Mortality	Co-Infections	Co-Infected Mortality (n/N)	Non-Infected Mortality (n/N)	Odds Ratio	95% CI	*p*-Value
**<48 h from Hospital Admission**							
Aissaoui et al. [44]	Hospital	COVID-19: 6/155	COVID-19: 5/6	COVID-19: 54/149	8.8	(1.001–77.26)	0.030
Doubravská et al. [50]	28-d	COVID-19: 46/171	COVID-19: 27/46	COVID-19: 49/125	2.2	1.11–4.39	0.025
Lee et al. [46]	ICU	Influenza: 32/117	Influenza: 7/32	Influenza: 18/85	1.04	(0.39–2.8)	1
Martin-Loeches et al. [51]	ICU	Influenza:451/2901	Influenza: 147/451	Influenza: 446/2233	1.94	(1.55–2.42)	<0.001
Schoettler et al. [43]	Hospital	COVID-19: 15/114 Influenza: 5/76	COVID-19: 6/15 Influenza: 2/5	COVID-19: 34/99 Influenza: 27/71	1.27 1.09	0.42–3.88 0.17–6.92	0.67 0.93
**<48 h from ICU admission**							
Chu et al. [49]	Hospital	COVID-19: 71/373 Influenza: 80/373	COVID-19: 21/71 Influenza: 10/80	COVID-19: 43/302 Influenza: 18/293	2.53 2.18	1.38–4.62 0.96–4.94	0.004 0.089
Aziza et al. [45]	30-d	Influenza: 55/130	Influenza: 14/55	Influenza: 16/75	1.26	(0.55–2.86)	0.674
Rouzé et al. [47]	28-d	COVID-19: 55/568 Influenza: 162/482	COVID-19: 24/55 Influenza: 47/162	COVID-19: 142/513 Influenza: 85/320	2.02 1.13	1.15–3.57 0.74–1.72	0.018 0.589
Bergmann et al. [48]	30-d	COVID-19: 68/289 Influenza: 8/39	COVID-19: 21/68 Influenza: no data	COVID-19: 40/221 Influenza: no data	2.02 No data	(1.09–3.75) No data	0.027 >0.05
Estenssoro et al. [52]	Hospital	Influenza: 80/337	Influenza: 40/80	Influenza: 150/245	0.63	0.38–1.05	0.089

**Table 4:** Pooled number and percentage of bacterial genera isolated from co-infected critically ill patients with COVID-19 and influenza, stratified by timing of co-infection diagnosis (<48 h from ICU admission vs. hospital admission). Data are presented as absolute counts (n) and percentages (%) relative to the total number of co-infected patients within each subgroup. Only studies reporting complete microorganism counts were included. Species-level data were aggregated at the genus level for consistency.

Genus	COVID-19 (N = 613)	COVID-19 <48 h ICU (N = 499)	COVID-19 <48 h Hospital (N = 114)	Influenza (N = 789)	Influenza <48 h ICU (N = 193)	Influenza <48 h Hospital (N = 596)
*Acinetobacter* spp.	2 (0.3%)	2 (0.4%)	0 (0%)	18 (2.3%)	2 (1.0%)	16 (2.7%)
*Bacteroides* spp.	0 (0%)	–	–	1 (0.1%)	0 (0%)	1 (0.2%)
*Burkholderia* spp.	0 (0%)	–	–	1 (0.1%)	1(0.5%)	–
*Chlamydophila* spp.	0 (0%)	–	–	3 (0.4%)	0 (0%)	3 (0.5%)
*Citrobacter* spp.	3 (0.5%)	1 (0.2%)	2 (1.8%)	0 (0%)	0 (0%)	–
*Enterobacter* spp.	11 (1.8%)	8 (1.6%)	3 (2.6%)	6 (0.8%)	3 (1.6%)	5 (0.8%)
*Enterococcus* spp.	14 (2.3%)	13 (2.6%)	1 (0.9%)	2 (0.3%)	2 (1.0%)	–
*Escherichia coli*	12 (2.0%)	10 (2.0%)	2 (1.8%)	22 (2.8%)	8 (4.1%)	14 (2.3%)
*Haemophilus influenzae*	12 (2.0%)	11 (2.2%)	1 (0.9%)	36 (4.6%)	19 (9.8%)	17 (2.9%)
*Klebsiella* spp.	25 (4.1%)	19 (3.8%)	6 (5.3%)	31 (3.9%)	4 (2.1%)	27 (4.5%)
*Legionella* spp.	0 (0%)	–	–	5 (0.6%)	0 (0%)	5 (0.8%)
*Moraxella catarrhalis*	4 (0.7%)	4 (0.8%)	–	1 (0.1%)	1 (0.5%)	–
*Morganella* spp.	1 (0.2%)	0 (0.0%)	1 (0.9%)	5 (0.6%)	4 (2.1%)	1 (0.2%)
*Mycobacterium* spp.	0 (0%)	–	–	3 (0.4%)	0 (0%)	3 (0.5%)
*Mycoplasma* spp.	0 (0%)	–	–	4 (0.5%)	0 (0%)	4 (0.7%)
*Nocardia* spp.	0 (0%)	–	–	1 (0.1%)	0 (0%)	1 (0.2%)
*Pneumocystis* spp.	0 (0%)	–	–	4 (0.5%)	0 (0%)	4 (0.7%)
*Proteus* spp.	9 (1.5%)	5 (1.0%)	4 (3.5%)	0 (0.0%)	–	–
*Pseudomonas aeruginosa*	15 (2.4%)	13 (2.6%)	2 (1.8%)	59 (7.5%)	0 (0%)	59 (9.9%)
*Serratia* spp.	8 (1.3%)	6 (1.2%)	2 (1.8%)	6 (0.8%)	1 (0.5%)	5 (0.8%)
*Shewanella* spp.	0 (0%)	–	–	1 (0.1%)	0 (0%)	1 (0.2%)
*Staphylococcus aureus*	66 (10.8%)	50 (10.0%)	16(14.0%)	120 (15.2%)	53 (27.5%)	67 (11.2%)
*Staphylococcus* spp.	1 (0.2%)	1 (0.2%)	–	6 (0.8%)	2 (1.0%)	4 (0.7%)
*Stenotrophomonas* spp.	4 (0.7%)	2 (0.4%)	2 (1.8%)	5 (0.6%)	1 (0.5%)	4 (0.7%)
*Streptococcus* spp.	33 (5.4%)	32 (6.4%)	1 (0.9%)	313 (39.7%)	63 (32.6%)	250 (41.9%)

**Table 5:** Methodology of COVID-19, influenza and bacterial co-infection diagnosis.

	COVID-19 + Influenza		Co-Infections	
	Sample	Method	Sample	Method
Aissaoui et al. (2022) [44]	Nasopharyngeal Swab—awake, Lower Respiratory Tract—intubated	RT-PCR	Blood, Lower Respiratory tract	Cultures
Aziza et al. (2021) [45]	Upper/lower respiratory track	PCR	Respiratory/blood	Cultures
Lee et al. (2022) [46]	Nasopharyngeal/pharyngeal swab	PCR	Respiratory/blood	Cultures
Martin-Loeches et al. (2017) [51]	Nasopharyngeal swab	PCR	Endotracheal aspirates/blood/pleural fluid	Cultures
Bergmann et al. (2023) [48]	No data	PCR	Sputum/endotracheal aspirates/BAL/blood; Urine	Cultures; Antigen test for *S. pneumoniae* & *L. pneumophila*
Chu et al. (2023) [49]	No data	PCR	Sputum/tracheal aspirate/BAL/Blood/pleural	Cultures
Doubravská et al. (2024) [50]	Nasopharyngeal/endotracheal	PCR	Blood/lower respiratory tract; Urine	Cultures, PCR for bacterial nucleic acid, serological methods for *mycoplasma* and *chlamydophila;* Antigen test for *S. pneumoniae* & *L. pneumophila*
Rouzé et al. (2021) [47]	Nasopharyngeal/respiratory secretions	PCR	Endotracheal aspirates/Blood; Urine	Cultures; Antigen test for *S. pneumoniae* & *L. pneumophila*
Estenssoro et al. (2010) [52]	Respiratory specimens	RT-PCR	Respiratory specimens	No data
Schoettler et al. (2023) [43]	Nasal/Throat swab/tracheal aspirate/Bal	PCR	Respiratory sample	No data

## Data Availability

Data are available from the authors upon reasonable request.
